# The revision and phylogenetic position of *Hippasa bifasciata* Buchar, 1997 (Araneae, Lycosidae)

**DOI:** 10.3897/BDJ.13.e166495

**Published:** 2025-08-21

**Authors:** Changjun Wu, Zehong Tao, Yang Wang, Yufa Luo

**Affiliations:** 1 Key Laboratory of Wetland Biodiversity of the Jianhu Basin of Shaoxing, School of Life and Environmental Sciences, Shaoxing University, shaoxing, China Key Laboratory of Wetland Biodiversity of the Jianhu Basin of Shaoxing, School of Life and Environmental Sciences, Shaoxing University shaoxing China

**Keywords:** new combination, morphology, taxonomy, DNA barcoding, phylogeny, Lycosinae

## Abstract

**Background:**

*Hogna* Simon, 1885 is the second-largest genus in the family Lycosidae after *Pardosa* C. L. Koch, 1847 (517 species), including 232 species so far. This genus has a cosmopolitan distribution spanning multiple continents. However, only four species (*Hogna rubetra* (Schenkel, 1963), *Hogna trunca* Yin, Bao & Zhang, 1996, *Hogna jiafui* Peng, Yin, Zhang & Kim, 1997 and *Hogna arborea* Lo, Wei & Cheng, 2023) have been recorded in China.

**New information:**

A new combination, *Hogna bifasciata* (Buchar, 1997), **comb. nov.** (from Yunnan and Sichuan Provinces in south-western China), is proposed with both morphological and molecular evidence. Detailed morphological descriptions, photographs, scanning electron micrographs and a distribution map are provided. This species is distinguished from congeners by the unique structure of the female epigyne and its somatic pattern. Molecular phylogenetic analyses suggest *H. bifasciata* (Buchar, 1997) and all analysed *Hogna* species cluster together within the subfamily Lycosinae and the species is sister to the group, including *Hogna frondicola* Emerton, 1885, *Hogna carolinensis* Walckenaer, 1805 and *Hogna crispipes* L. Koch, 1877.

## Introduction

The genus *Hogna* Simon, 1885 is the second largest genus in the family Lycosidae Sundevall, 1833 and exhibits a global distribution spanning multiple continents, with notably high species diversity in South Africa, the United States, Brazil, Australia and other regions. Its range extends across tropical to temperate zones, including adaptations to island environments. Currently, *Hogna* includes 232 described species, with four species reported from China, such as *Hogna rubetra* (Schenkel, 1963) (♀, the detailed type locality has not been recorded), *Hogna trunca* Yin, Bao & Zhang, 1996 (♂/♀, Zhejiang), *Hogna jiafui* Peng, Yin, Zhang & Kim, 1997 (♂, the detailed type locality has not been recorded) and *Hogna arborea* Lo, Wei & Cheng, 2023 (♂/♀, Taiwan), three of which are endemics (Brignoli 1983, Song et al. 1999, Shida 2022, Lo et al. 2023, World Spider Catalog 2025).

Morphological and DNA barcoding data of the spiders collected from Yunnan and Sichuan of China, suggest that *Hippasa bifasciata* Buchar, 1997 (Buchar, 1997) can be transferred to the genus *Hogna*. We provide comprehensive morphological descriptions and illustrations of *Hogna bifasciata* (Buchar 1997).

## Materials and methods

### Morphological treatment

Specimens were collected from Yunnan and Sichuan Provinces of China by hand. All materials were fixed and preserved in absolute ethanol at −20°C. Morphological examinations and imaging were conducted using a Phenix stereomicroscope. Epigynes of female specimens were dissected, cleared in pancreatin solution (Álvarez-Padilla and Hormiga 2007) and photographed with a Sony digital camera mounted on a Wemacro focus-stacking system (2021). Image stacks were processed using Helicon Focus 3.10. Samples were air-dried, sputter-coated with gold and imaged with a Zeiss SIGMA 300 field-emission Scanning Electron Microscope (SEM). All measurements of morphological structures are given in millimetres (mm) and were obtained using the Phenix stereomicroscope. Ocular diameters represent maximum width. Total body length excludes chelicerae and spinnerets. Leg segment lengths comprise: total length (femur, patella and tibia, metatarsus, tarsus).

The following abbreviations are employed: **ALE** anterior lateral eye; **AME** anterior median eye; **AME–AME** distance between AMEs; **AME–ALE** distance between AME and ALE; **CD** copulatory duct; **MS** median septum; **PLE** posteriorlateral eye; **PME** posterior median eye; **PME–PME** distance between PMEs; **PME–PLE** distance between PME and PLE; **S** spermathecae.

All examined specimens are deposited in the Key Laboratory of Wetland Biodiversity of the Jianhu Basin of Shaoxing, School of Life and Environmental Sciences, Shaoxing University, Shaoxing, China.

### DNA extraction, amplification and sequencing

Genomic DNA extraction from two legs of the specimen (LZ_102) of *H. bifasciata* (Buchar, 1997) was performed using the TIANamp Genomic DNA Kit (Tiangen, Beijing, China). Partial sequences of the mitochondrial cytochrome c oxidase subunit I (*COI*), 12S rRNA and 16S rRNA genes were amplified with primer pairs: for *COI*, LCO1490 (5′-GGTCAACAAATCATAAAGATATTGG-3′) / HCO2198 (5′-TAAACTTCAGGGTGACCAAAAAATCA-3′) (Folmer et al. 1994); for 12S rRNA, 12Sai (5′-AAACTAGGATTAGATACCCTATTAT-3′) / 12Sbi (5′-AAGAGCGACGGGCGATGTGT-3′) (Kocher et al. 1989); and for 16S rRNA, 16Sar (5′-CGCCTGTTTATCAAAAACAT-3′) / 16SB2（5′-CTCCGGTTTGAACTCAGATCA-3′）(Tan et al. 1999). PCR reactions (25 μl volume) contained: 12.5 μl MyFi Mix (Bioline, USA), 1 μl each primer (10 μM), 2 μl DNA template and 8.5 μl ddH₂O. For *COI* amplification, the PCR protocol was as follows: initial denaturation at 94°C for 5 min; 40 cycles of 94°C (30 s), 45°C annealing (90 s) and 72°C extension (1 min); final extension at 72°C for 5 min. For 12S rRNA amplification, the PCR protocol was as follows: 94°C for 1 min; 35 cycles of 94°C (25 s), 49°C (30 s), 72°C (20 s); 72°C for 6 min. For 16S rRNA amplification, the PCR protocol was as follows: 94°C for 1 min; 35 cycles of 94°C (30 s), 49°C (45 s), 72°C (1 min); 72°C for 5 min. Bidirectional Sanger sequencing of purified amplicons was performed at Tsingke Biotechnology Co., Ltd., Hangzhou, China. New sequences were submitted to GenBank (Suppl. material 1).

### Phylogenetic reconstruction

First, the concatenated *COI*, 12S rRNA and 16S rRNA dataset (Suppl. material 2) of 329 Lycosidae species, including the sequences of 328 species derived from GenBank and BOLDSYSTEM (Suppl. material 1) was used to reconstruct the Maximum Likelihood (ML) tree in W-IQ-TREE (Trifinopoulos et al. 2016). The FreeRate heterogeneity and Bayesian Information Criterion identified the optimal substitution model for each gene partition (GTR + F + R6 for *COI*; GTR + F + I + G4 for 12S rRNA; GTR + F + R4 for 16S rRNA). Perturbation strength (p) and number of iterations since the last optimal tree discovery (c) were specified as 0.5 and 1000, respectively. Branch support was assessed via ultrafast bootstrap (UFBoot; Minh et al. (2013)) and SH-aLRT (Guindon et al. 2010) with 1,000 maximum replicates and a minimum correlation coefficient threshold of 0.99. *Pisaura ancora* Paik, 1969, *Pisaura lama* Bösenberg & Strand, 1906 and *Pisaura mirabilis* Clerck, 1757 were used as outgroups. Second, a phylogeny of Lycosinae and Pardosinae was reconstructed using the concatenated *COI*, 12S rRNA and 16S rRNA sequences from 106 species of Lycosinae and Pardosinae (Suppl. material 3) with the outgroups *Hippasa pantherine* Pocock, 1899, *Hippasa deserticola* Simon, 1889 and *Hippasa madraspatana* Gravely, 1924. This analysis was also executed in W-IQ-TREE with the same sets as above, using the partition-specific substitution models (GTR+F+I+G4 for *COI*; TIM2+F+I+G4 for 12S rRNA; and TIM2+F+R3 for 16S rRNA).

## Taxon treatments

### Hogna bifasciata

(Buchar, 1997)

4B8D5D24-64CA-51D9-9795-086EFC841EAB

Hippasa bifasciata Buchar, 1997: 12, f. 10-14, (♀)
Hogna bifasciata (Buchar, 1997) comb. nov.

#### Materials

- **Type status:** Other material. **Occurrence:** recordedBy: Changjun Wu; individualCount: 1; sex: 1 female; lifeStage: 1 adult; occurrenceID: 0CB83E23-3AB5-5908-82E4-060480E49B76; **Taxon:** namePublishedInID: Buchar, J. 1997. Lycosidae aus Bhutan 1. Venoniinae und Lycosinae (Arachnida, Araneae). Entomologica Basiliensis 20: 5-32.; scientificName: *Hogna bifasciata* (Buchar, 1997); order: Araneae; family: Lycosidae; genus: Hogna; specificEpithet: *bifasciata*; scientificNameAuthorship: Wu, Tao & Luo; **Location:** continent: Asia; country: China; countryCode: CHN; stateProvince: Yunnan; county: Eshan Yi Autonomous; municipality: Yuxi; locality: Linjiang Park; verbatimElevation: 1536 m; decimalLatitude: 24.172778; decimalLongitude: 102.406389; georeferenceProtocol: GPS; **Identification:** identifiedBy: Yufa Luo; dateIdentified: 12-2024; **Event:** samplingProtocol: by hand; year: 2024; month: 8; day: 18; **Record Level:** basisOfRecord: PreservedSpecimen
- **Type status:** Other material. **Occurrence:** recordedBy: Haodong Chen; individualCount: 1; sex: 1 female; lifeStage: 1 adult; occurrenceID: 15208CC4-F6AF-54B6-B91F-E9E5A6031EAF; **Taxon:** namePublishedInID: Buchar, J. 1997. Lycosidae aus Bhutan 1. Venoniinae und Lycosinae (Arachnida, Araneae). Entomologica Basiliensis 20: 5-32.; scientificName: *Hogna bifasciata* (Buchar, 1997); order: Araneae; family: Lycosidae; genus: Hogna; specificEpithet: *bifasciata*; scientificNameAuthorship: Wu, Tao & Luo; **Location:** continent: Asia; country: China; countryCode: CHN; stateProvince: Sichuan; county: Liangshan Yi Autonomous Prefecture; municipality: Xichang; locality: Taihe Town, Yanshui Village; verbatimElevation: 1471.5 m; decimalLatitude: 27.881389; decimalLongitude: 102.161667; georeferenceProtocol: GPS; **Identification:** identifiedBy: Yufa Luo; dateIdentified: 12-2024; **Event:** samplingProtocol: by hand; year: 2023; month: 8; day: 11; **Record Level:** basisOfRecord: PreservedSpecimen

#### Description

**Female** Total length 8.06; carapace 3.52 long, 2.51 wide; abdomen 4.50 long, 2.38 wide. Eye sizes and interdistances: AME 0.13, ALE 0.11, PME 0.30, PLE 0.22, AME–AME 0.10, AME–ALE 0.04, PME–PME 0.41, PME–PLE 0.42. Clypeus height 0.13. Leg measurements: Ⅰ 9.85 (2.84, 3.59, 1.95, 1.47); Ⅱ 9.25 (2.68, 3.25, 1.97, 1.35); Ⅲ 8.59 (2.40, 2.80, 2.12, 1.27); Ⅳ 13.59 (3.54, 4.18, 4.15, 1.72). Leg formula: 4-1-2-3.

**Figure 1.**
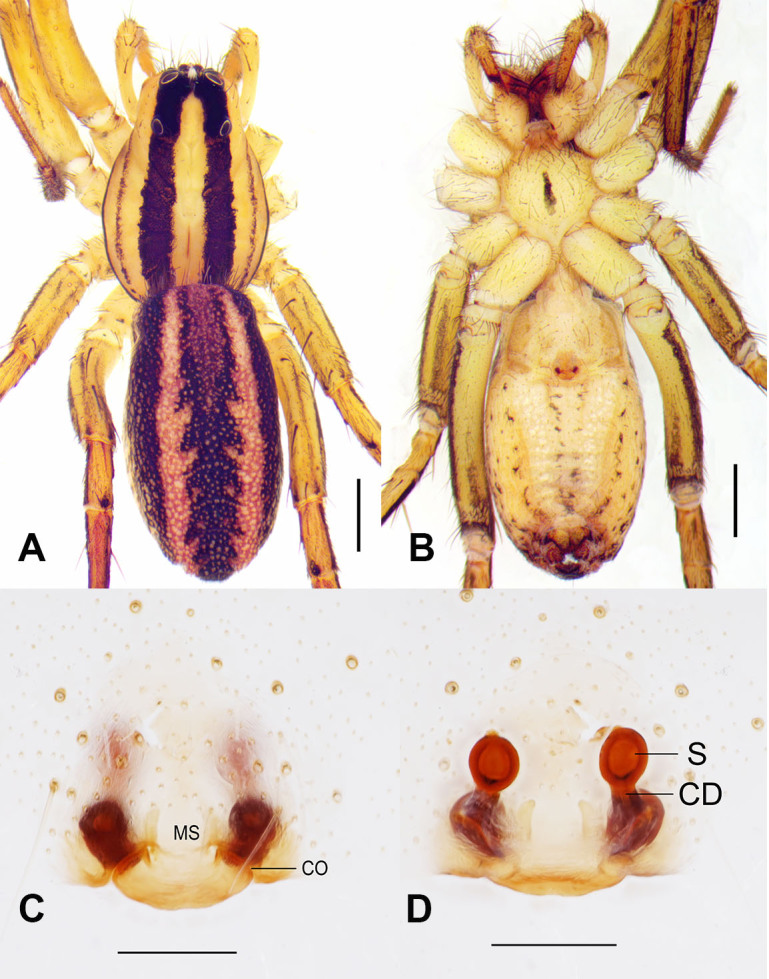
*Hogna bifasciata* (Buchar, 1997). **A** Habitus, dorsal view; **B** Same, ventral view; **C** Epigyne, ventral view; **D** Vulva, dorsal view. Abbreviations: CD = copulatory duct, CO = copulatory opening, MS = median septum, S = spermathecae. Scale bars: 1 mm (A, B), 0.2 mm (C, D).

Colouration (Fig. 1A, B): Carapace ground colour pale yellow; median band yellowish-brown, flanked by two prominent black longitudinal stripes. Each stripe accompanied by a black lateral patch. Cervical groove and radial furrow distinct; fovea longitudinal, rust-red. Ocular region pale yellow, PME and PLE situated on black longitudinal stripes, PME interocular space bearing a distinctive cluster of white setae. Chelicerae yellow, each with a central black stripe prolaterally, surface setose; with three promarginal teeth and three retromarginal teeth. Labium dark yellow, width exceeding length. Endites yellow, length exceeding width. Sternum cordate, pale yellow, densely covered with black macrosetae; central longitudinal black stripe present. Abdomen oval, dorsum predominantly black with a distinct lanceolate cardiac mark, reddish-brown, flanked by two rust-pink longitudinal stripes laterally. Ventral surface yellow with scattered black spots.

**Figure 2.**
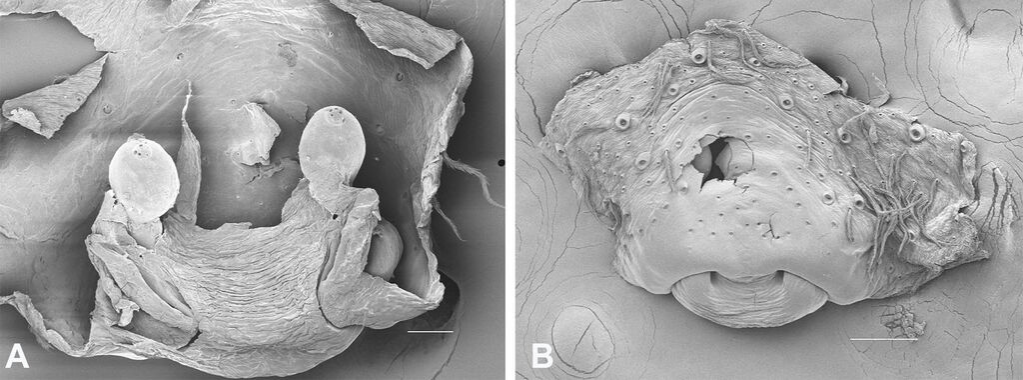
*Hogna bifasciata* (Buchar, 1997). **A** Epigyne, dorsal view; **B** Same, ventral view. Scale bars: 0.05 mm (A), 0.1 mm (B).

Epigyne (Fig. 1C, D, Fig. 2A, B): Epigynal plate longer than wide, with defined margins; median septum (MS) present, atrium absent. Spermathecae (S) conspicuous, bulbous sac-like structures, distinctly separated. Copulatory ducts (CD) connecting copulatory openings to spermathecae, coiled.

**Male.** Unknown.

**DNA barcodes**:

5'-TCGGCCATAATAGGGACGGCTATAAGAGTATTGATTCGTATAGAATTAGGTAATCCTGGAAGTTTATTAGGAGATGATCATTTGTATAATGTAATAGTTACTGCTCATGCTTTTGTTATGATTTTTTTTATAGTAATGCCAATTCTTATTGGTGGTTTTGGAAATTGATTAGTGCCTTTGATATTAGGGGCTCCTGATATATCATTTCCTCGTATAAATAATCTTTCTTTTTGATTATTACCTCCTTCTTTGTTTTTATTATCAATGTCTTCTATAGTAGAGATAGGGGTAGGGGCAGGATGAACTGTTTATCCTCCTTTGGCTTCTAGAATAGGTCATATAGGGAGTTCTATAGATTTTGCTATTTTTTCTTTGCATTTAGCTGGGGCTTCTTCTATTATAGGTGCGGTTAATTTTATTTCTACTATTATTAATATACGTATATTAGGTATGACTATAGAGAGGGTTCCTCTATTGGTTTGATCAGTATTAATTACTGCTGTTTTGTTATTACTTTCTTTACCTGTGTTAGCGGGTGCTATTACTATGTTGTTGACAGATCGTAATTTTAATACTTCTTTTTTTGATCCGGCGGGAGGAGGAGATCCTATTTTGTTTCAGCATTTATTTTGATT-3'

(**molecular specimen, LZ_102; female from Yuxi; partial sequence of *COI*; GenBank accession number: PV917064**).

5'-AAAACCTATTTATATCGGCGGCATTTCATCTTATTAGAGGAACCTGTTCTTTAATCGATAATCCACGTTAAATTTCACTTTAATTAAAAATTTATATACCGCCATCTAAAAAACTAATATAAAAATAATATTTTAAATAAAAAATAAAAAGTTAGGTCAAGGTGTAATCTACATTCAAGAAACAATGGGTTACATTAAATATAATTTAAGAATTTTAATATAAAAACATTTTTTGAAAAAGGATTTAAAAGTAATAATTAAATAATATTTAATTATGATTAAGATATAAATGTGCACACATCGCCCGTCGCTCTT-3'

(**molecular specimen, LZ_102; female from Yuxi; partial sequence of 12S rRNA; GenBank accession number: PV917103**).

5'-ATGAATATTAAATAGCCGCTTTTGTGCTAAGGTAGCATAATAATTTGTCTTTTAATTAAAGACTAGAACAAAAGACTTAACATCTCAATTAAATTTTTAGGAAAAACAATTTAAATTATCTTAATTGTAAAAAGACAATTATACAATAGATAGACGATAAGACCCTATCGAACTTTACTTTAGTTTAACTGGGGCAGTTAATTAATTATTATTTTAAAAAAAATAAATTTTCAATTTGACCTAATATTATTAATAAAATAATTAAGTTACCGTAGGGATAACAGCGTAATAAAATTTATAAGACCTTATTTAAAATTAAGATTGCGACCTCGATGTTGATTTAATATCCTACTTTACGCAATAGTAAATTAAGGAAGTCTGTTCGACTTTTAAAAAATTACATGATCTGAGTT-3'

(**molecular specimen, LZ_102; female from Yuxi; partial sequence of 16S rRNA; GenBank accession number: PV917102**).

**Phylogeny**

**Figure 3.**
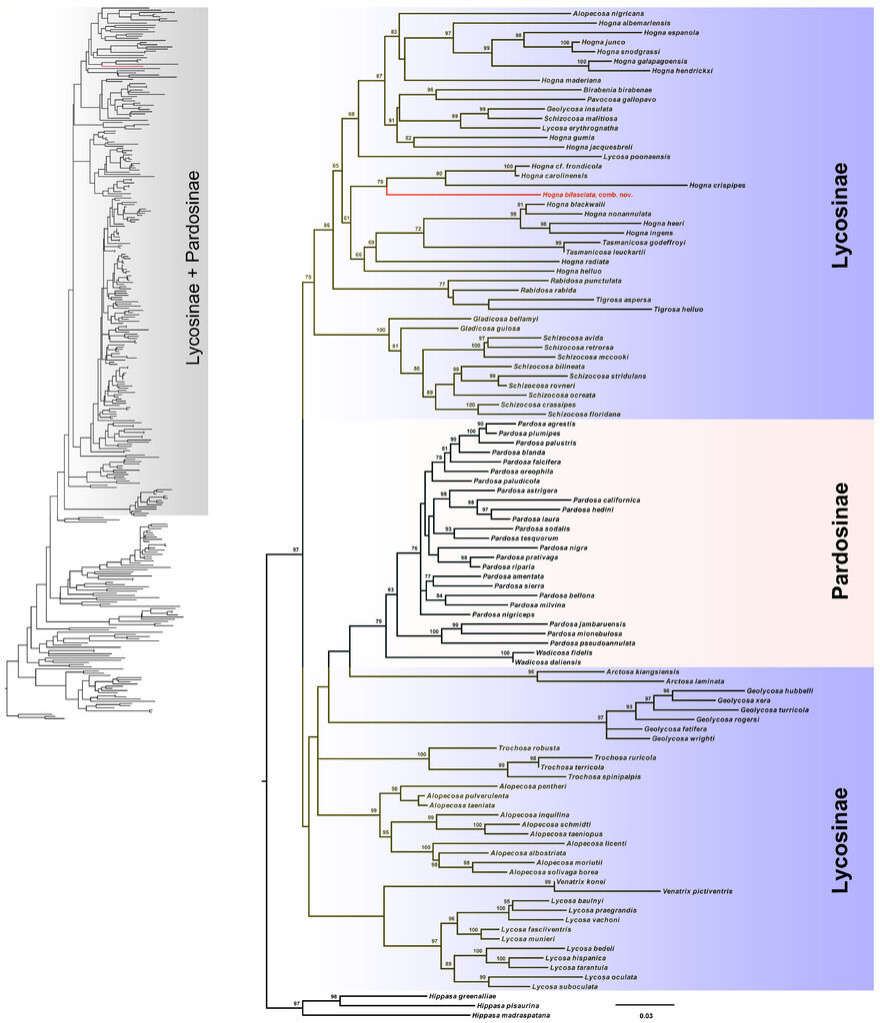
Phylogenetic tree reconstructed using the Maximum Likelihood method. **A** The Maximum Likelihood tree was reconstructed using the concatenated *COI*, 12S rRNA and 16S rRNA sequences of 329 species of Lycosidae; **B** The Maximum Likelihood tree was reconstructed using the concatenated *COI*, 12S rRNA and 16S rRNA sequences of 106 species of Lycosinae and Pardosinae. The numbers at the nodes represent bootstrap support values from the Maximum Likelihood analyses. The species *Hogna bifasciata* (Buchar, 1997) is shown with red colour in the phylogenetic trees.

The aligned data had lengths of 635 bp for *COI*, 315 bp for 12S rRNA and 413 bp for 16S rRNA. The ML tree (Fig. 3) places *Hogna bifasciata*, comb. nov. into the subfamily Lycosinae. Moreover, the species and all analysed *Hogna* species, including the type species *Hogna radiata* Latreille, 1817 clustered into a clade. *Hogna bifasciata* (Buchar, 1997) is sister to the group including *Hogna frondicola* Emerton, 1885, *Hogna carolinensis* Walckenaer, 1805 and *Hogna crispipes* L. Koch, 1877.

#### Diagnosis

Females of the species *Hogna bifasciata* (Buchar, 1997) resemble *Hogna graeca* Roewer, 1951 in carapace colouration and markings (compare fig. 1386 in Koch (1847)), but can be distinguished from the latter by a distinct cluster of white setae between the posterior median eyes and a median longitudinal black stripe on the sternum. While sharing the bulbous spermathecae with *Hogna cinica* Tongiorgi, 1977 (compare figs. 73–84 in Sherwood et al. (2023)), *Hogna bifasciata* (Buchar, 1997) differs in spermathecal orientation — the spermathecae are subparallel (versus medially opposed in *H. cinica*). In addition, the species exhibits the unique traits with an epigynal plate longer than wide, globose spermathecae and coiled copulatory ducts.

#### Distribution

**Figure 4.**
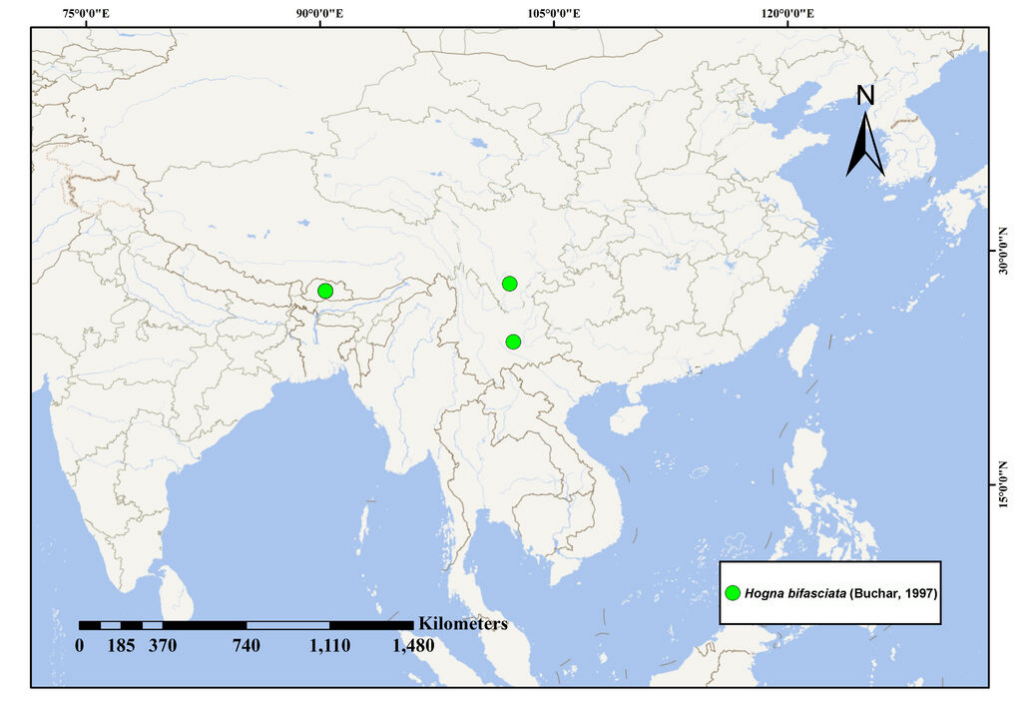
Geographic distribution of *Hogna bifasciata* (Buchar, 1997).

Currently known from Bhutan and the Yunnan and Sichuan Provinces of China (Fig. 4).

## Discussion

The taxonomic boundaries of *Hogna* remain contentious due to morphological overlap with *Lycosa* Latreille, 1804 and *Geolycosa* Montgomery, 1904 (Murphy et al. 2006, Planas et al. 2013, Piacentini and Ramírez 2019, Lo et al. 2023). Compounding this uncertainty, Brady (2012) demonstrated that genital morphology — traditionally pivotal for lycosid taxonomy — fails to reliably distinguish *Hogna* from other Lycosinae genera, instead proposing carapace patterns and ocular arrangements as diagnostic traits. This diagnostic ambiguity, coupled with historically inadequate descriptions, suboptimal illustrations and lost type specimens of many early-described species, has rendered *Hogna* a taxonomic "dumping ground" for large-bodied Lycosinae of unresolved affinity. Consequently, the genus lacks clear boundaries, impeding biogeographic reconstructions such as tracing the origins of putative coloniser species on the Madeira Archipelago (Brady 2012).

Despite these challenges, integrative evidence supports the placement of *H. bifasciata* (Buchar, 1997) within *Hogna*: mitochondrial phylogeny (*COI*, 12S rRNA and 16S rRNA) clusters it within a *Hogna*-affiliated clade, distinct from *Lycosa*/*Geolycosa*, while morphology aligns with Brady’s (Brady 2012) criteria. This affirms its generic assignment despite current limitations in female-only material and the non-monophyletic status of the genus.

## Supplementary Material

XML Treatment for Hogna bifasciata

E307DE7D-6652-58CA-AA84-52E9723B1D74 10.3897/BDJ.13.e166495.suppl1 Supplementary material 1 Samples used in this study taxon name, specimen voucher, sample collection locality and GenBank accession numbers Data type Table Brief description The sample information of 329 species which were used to reconstruct the Maximum Likelihood (ML) tree in W-IQ-TREE. File: oo_1380628.xlsx https://binary.pensoft.net/file/1380628 Yang Wang, Yufa Luo

F8B27BD4-74DA-5689-A33F-24C9B0AC735F 10.3897/BDJ.13.e166495.suppl2 Supplementary material 2 The concatenated *COI*, 12S rRNA and 16S rRNA dataset of 329 Lycosidae species Data type Sequence dataset Brief description The concatenated *COI*, 12S rRNA and 16S rRNA dataset of 329 Lycosidae species was used to reconstruct the Maximum Likelihood (ML) tree in W-IQ-TREE. File: oo_1380630.fas https://binary.pensoft.net/file/1380630 Yang Wang, Yufa LuoYang Wang, Yufa Luo

2E5AC622-DE0B-5F12-9490-7DA255D34F22 10.3897/BDJ.13.e166495.suppl3 Supplementary material 3 The concatenated *COI*, 12S rRNA and 16S rRNA dataset of 106 Lycosinae and Pardosinae species Data type Sequence dataset Brief description The concatenated *COI*, 12S rRNA and 16S rRNA dataset of 106 Lycosinae and Pardosinae species was used to reconstruct the Maximum Likelihood (ML) tree in W-IQ-TREE. File: oo_1380631.fas https://binary.pensoft.net/file/1380631 Yang Wang, Yufa Luo
